# Psychological interventions for refugees with depression: a systematic literature review

**DOI:** 10.1186/s12888-024-06447-y

**Published:** 2025-01-08

**Authors:** Cornelia Uhr, Silke Pawils, Nexhmedin Morina, Heba Alkailani, Franka Metzner

**Affiliations:** 1 Department of Medical Psychology, University Medical Centre Hamburg-Eppendorf, Hamburg, Germany; 2 Institute of Psychology, University of Münster, Münster, Germany; 3 Educational Science with a focus on Special Education (“Emotional and Social Development”), University of Siegen, Siegen, Germany

**Keywords:** Refugees, Asylum seekers, Depression, Psychological intervention, Group intervention

## Abstract

**Background:**

Ongoing global crises are forcing an increasing number of people to seek refuge in other countries. Refugees have often experienced multiple potentially traumatic events before and during their flight and are burdened by psychosocial problems in exile. Epidemiological research suggests that many refugees suffer from depression and need psychological care. Yet, a systematic review of psychological interventions for refugees with depression is lacking.

**Method:**

After registering in the International Prospective Register of Systematic Reviews (PROSPERO), a systematic search for trials of psychological interventions for adult refugees with depression was conducted across three electronic databases (MEDLINE, Web of Science, & PsycINFO). Relevant data reported in original journal publications were extracted, synthesized and assessed qualitatively by two independent raters. The methodological quality of included trials was assessed.

**Results:**

Of 1316 publications, a total of 20 studies met eligibility criteria. Nine of these trials were carried out in an individual setting and ten in a group setting, with one of the trials being conducted digitally. Nine studies were designed as a randomized controlled trial (RCT), with only one of them using an active control group. In nine trials, the use of an interpreter was reported. Three of the trials applied multimodal treatments, and a total of sixteen studies applied manualized treatments. Seventeen interventions were adaptations of treatment programs developed in high income countries within a western context. Overall, nineteen out of twenty trials reported a significant improvement in depressive symptoms. Culturally adapted cognitive behavioural therapy (CA-CBT) was most frequently used (4 RCTs) and produced large effect sizes. Overall, all trials had limitations in study design.

**Conclusions:**

Our current review suggests that psychological interventions, and in particular CA-CBT interventions, can significantly improve depressive symptoms in refugees. However, the small number of trials and limitations in study design underscore the need for more research in this field.

The protocol for this review was registered in PROSPERO; registration number: CRD42021251943.

## Background

According to the United Nations High Commissioner for Refugees (UNHCR) [1], there was a global total of 108.4 million forcibly displaced persons at the end of 2021. Refugees often report numerous potentially traumatic experiences before they arrive in exile [2, 3]. Research on refugee mental health has therefore focused heavily on post-traumatic stress disorder (PTSD), which is very prevalent in this population [4]. Yet, depression is similarly prevalent in refugees [5–7]. Literature reviews by Turrini et al. [4] and Lindert et al. [8] revealed that up to 44% of asylum seekers and refugees suffer from depression. Importantly, both pre-migration traumatic experiences as well as migration itself make migrants vulnerable to depression [9]. Depression is a severe mental condition, which may significantly impair both language acquisition and social participation, and as such hinder integration into the host society. In addition to the individual suffering of refugees, the host society also faces increased costs if depression remains untreated, for example due to extended payment of social benefits [10].

Despite the high prevalence of depression in refugees, existing research on the efficacy of psychological interventions for refugees has mainly focused on reducing PTSD symptoms [4, 11]. Meta-analyses on the treatment of PTSD suggest that psychological interventions, and trauma-focused interventions in particular, are effective in reducing PTSD in refugees [12–16]. Furthermore, a subset of these trials suggests that interventions aimed at reducing PTSD are also effective in reducing comorbid depressive symptoms [15, 17].

The only published meta-analysis that explicitly covered refugees suffering from a depressive disorder is from Kip et al. [15]. This systematic review of randomized controlled trials (RCTs) on the efficacy of psychological interventions for PTSD and depression in refugees did not identify any trial focusing on depression alone. Kip et al. [15] found information about the percentage of patients with major depression in only four out of a total of 14 included trials [18–21], yielding a mean rate of 88.98%. Buhmann et al. [19] investigated the efficacy of Cognitive Behavioural Therapy (CBT), whereas Adenauer et al. [18], Hensel-Dittmann et al. [20] and Stenmark et al. [21] examined the efficacy of Narrative Exposure Therapy.

Overall, a recent overview of systematic reviews on mental health among refugees [11] found no systematic review focusing mainly on refugees with a clinically relevant depression.

Against this background, we aimed at systematically exploring current psychological intervention studies for clinical depression in refugees. We focused on both controlled and uncontrolled studies conducted with patients who met criteria for a depressive disorder.

## Materials and methods

Our methodological approach is based on the guidelines for the implementation and analysis of systematic reviews [22–25], which describe the research process and tools to summarize evidence relevant for decision-makers. This study also followed the Preferred Reporting Items for Systematic reviews and Meta-Analyses (PRISMA) 2020 statement [26] , including the recommended checklist for the publication of systematic reviews. The protocol for this review was registered in the International Prospective Register of Systematic Reviews (PROSPERO; registration number: CRD42021251943) after piloting the study selection process and before formal screening of search results against eligibility criteria.

### Inclusion and exclusion criteria

The inclusion and exclusion criteria were selected according to the Population, Intervention, Comparison, Outcome, and Study design (PICOS) format [24], see Table 1. The main structured research question describing the PICOS scheme was defined as “In adult refugees with depression (P), do psychological interventions (I), independent of a control group (C) improve depressive symptoms (O) in a pre-post study design (S)?”.

**Table 1:** Inclusion and exclusion criteria based on PICOS scheme

Patient / Population	
IC 1.1	Adults (> = 18 years old)
IC 1.2	Any depressive disorder at baseline (depression must be one inclusion criterion or the number of participants with depression must be given as a percentage). Comorbid diagnoses are accepted. Depression has been measured with a measure of depression based on the ICD or DSM
IC 1.3	Refugees or asylum seekers or undocumented migrants (definition according to International Organization for Migration (IOM))
EC 1	Internally displaced persons, other migrants
**Intervention**	
IC 2.1	Psychological intervention such as • CBT: psychological therapies that use cognitive, behavioural or cognitive behavioural techniques • Behaviour therapies • Integrative therapies, like IPT • Psychodynamic therapy • Systemic therapy • Other psychological interventions, e.g. counselling, psychoeducation, training All treatment modalities were considered for inclusion, such as face-to-face or online
IC 2.2	The intervention focuses on depression
EC 2	Primary focus on PTSD or mainly trauma exposition
**Comparator**	
IC 3	Not relevant (a comparator does not have to be present)
**Outcome**	
IC 4	Primary outcome: improvement in the severity of symptoms of depressive episodes/disorders • recording of symptoms according to ICD or DSM
**Publication**	
**IC 5**	Original studies published as peer reviewed journal articles with abstract and title in German or English language; no language restrictions in full text
**EC 5.1**	Unpublished studies, book chapters, congress contributions, doctoral theses
**EC 5.2**	Same sample analysed in two or more publications
**EC 5.3**	Full text not available
EC 5.4	Relevant results not presented in the full text
**Study design**	
IC 6	Study design of an intervention study: • pre-post comparison sufficient; control group does not have to exist

*PICOS* Population Intervention Comparison Outcome and Study design (PICOS), *CBT* Cognitive behavioural therapy, *IPT* Inter personal therapy, *PTSD* Post-traumatic-stress disorder, *ICD* International classification of diseases, *DSM* Diagnostic and statistical manual of mental disorders

In accordance with the International Organization for Migration [27], we defined the key terms as follows: *Refugees* as individuals who fled their country of origin to escape persecution for reasons of race, religion, nationality, membership of a particular social group or political opinion; *asylum-seekers* as those who left their country and are seeking refugee status in another country and awaiting a response to this claim; and *undocumented migrants* as individuals who migrated through irregular channels (i.e., movement outside of regulatory norms without the necessary authorization or documents required under immigration regulations) or who remained in a country without authorization or documents required under immigration regulations [27]. We applied the following inclusion criteria : (a) Being older than 17 years old and being a refugee or asylum seeker or undocumented migrant in a host country; (b) Meeting criteria for a diagnosis of depression as measured by an instrument based on the Diagnostic and Statistical Manual of Mental Disorders (DSM) [28] or International Classification of Diseases (ICD) [29]. Studies with participants with comorbid mental disorders were included; (c) Psychological interventions were applied to treat depression. We considered both face-to-face and online treatment modalities.

### Information sources

A search of peer-reviewed primary literature was conducted in October 2024 in the scientific databases Medline, PsycINFO, and Web of Science. There was no restriction on the publication period, but publications needed to have an abstract in English or German. 

### Search strategy

Before starting the search process, preliminary searches were carried out. The search string was tested in the database Medline to optimize our methodology and focus. In addition, we searched the PROSPERO database for any ongoing systematic review on our topic, yet we found none. For our final search string see Table 2.

**Table 2:** Terms used in systematic database literature search

Category	English language
A: Depression	depress* or MDD
B: Refugee	refugee* or asylum seeker* or evacuee* or displaced person* or displaced people
C: Intervention	treatment* or intervention* or therapy or psychotherapy or counselling* or trial* or training*

### Screening and study selection

Citations identified from the systematic search were exported to the reference management tool Citavi 6. Duplicates were removed and two independent reviewers, Cornelia Uhr (CU) and Franka Metzner (FM) screened all titles and abstracts using the inclusion and exclusion criteria, labelling excluded references with the reason for exclusion [23]. Publications labelled as “excluded” by both authors were removed, while publications that received conflicting votes (ineligible vs. potentially or probably eligible) were discussed until consensus was reached. The agreement rate was measured by determining the percentage of the sum of all matching “included” and “excluded” references, where the total number of all double screened references represented 100%. Interrater reliability was calculated for the studies using Cohen’s Kappa.

### Data extraction and synthesis

The selection of characteristics to be extracted from the included primary studies was discussed with the research team, CU and FM, and unanimous agreement was reached. After reviewing the full texts against the inclusion criteria, k = 20 studies were included into the systematic review. Using a structured table for data synthesis, relevant information was coded for describing the studies included.

### Assessment of methodological study quality and risk of bias

Two reviewer, CU and FM, independently rated the quality of the included trials by using the Mixed Method Appraisal Tool (MMAT) [30]. The MMAT was chosen because it is designed for mixed methods review and includes studies without a control condition. Out of five possible study types assessable using the MMAT, only the categories “Quantitative randomized controlled trials” and “Quantitative non-randomized trials” were used for the current literature review. The quality criteria were each rated as “met”, “not met” or “not enough information available”, and methodological study quality was subsequently rated as “high”, “medium” or “low. For a rating of “high” methodological study quality all quality criteria had to be met. A study was rated as having “medium” methodological quality if 3–4 quality criteria were met. A rating of “low” methodological quality was given if 0–2 quality criteria were met. Conflicting assessments between reviewers were discussed until consensus was reached and/or an independent third reviewer was asked when there was no consensus.

## Results

### Study selection

**Fig. 1:**
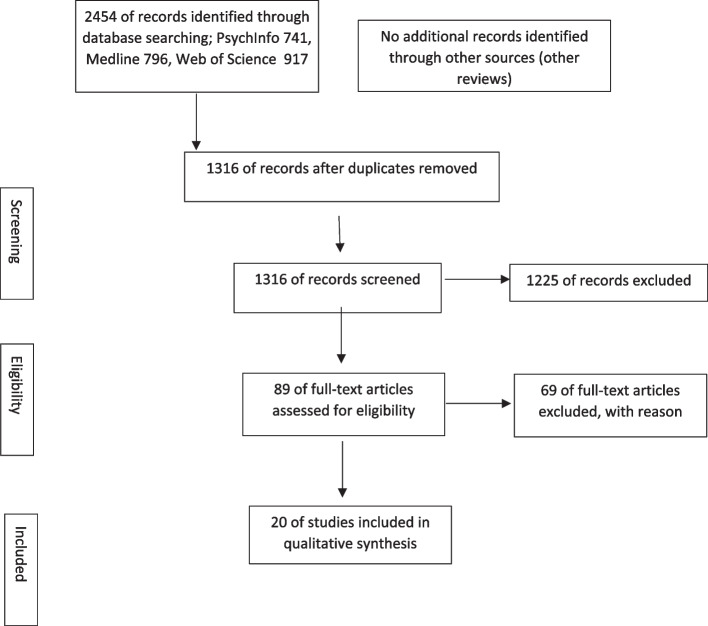
PRISMA-Flow-Diagram for the presentation of study selection process following Moher et al. (2009)

The study selection process is shown in Figure 1. After removing duplicates (k=1138 hits), two independent researchers screened titles and abstracts of the remaining k=1316 hits (agreement rate: 0,92%, interrater reliability Cohen`s Kappa = 0,43); k=1225 hits were excluded. The full texts of the remaining 89 publications were screened by two independent researchers to assess eligibility (agreement rate: 0,76%, interrater reliability Cohen`s Kappa = 0,5), 69 studies were excluded after full text screening (see Table 3). Consensus was always possible during the screening of title and abstract or full-text screening, so it was not necessary to ask independent third reviewers for their vote. All available abstracts were in English and no publication had to be excluded due to the language criterion.

**Table 3:** Overview of excluded studies with reason for exclusion (k = 69)

Citation	Reason for exclusion	Respec-tive PICOS criteria
Acarturk, C.; Konuk, E.; Cetinkaya, M.; Senay, I.; Sijbrandij, M.; Gulen, B.; Cuijpers, P. (2016): The efficacy of eye movement desensitization and reprocessing for post-traumatic stress disorder and depression among Syrian refugees: results of a randomized controlled trial. In: Psychological Medicine 46 (12), S. 2583–2593. https://doi.org/10.1017/S0033291716001070	Primary focus on PTSD	EC 2
Acarturk, Ceren; Konuk, Emre; Cetinkaya, Mustafa; Senay, Ibrahim; Sijbrandij, Marit; Cuijpers, Pim; Aker, Tamer (2015): EMDR for Syrian refugees with posttraumatic stress disorder symptoms: Results of a pilot randomized controlled trial. In: Eur J Psychotraumatol 6. https://doi.org/10.1037/t00742-000	Primary focus on PTSD or trauma exposition	EC 2
Ahmad, J., Okwuowulu, C., Sanusi, B., Bello, S. A., Talabi, F. O., Udengwu, N., & Gever, V. C. (2022). Impact of social media-based dance therapy in treating depression symptoms among victims of Russia-Ukraine war. Health Promotion International, 37(6). https://doi.org/10.1093/heapro/daac172	Not only refugees and asylum seekers	IC 1.3
Akhtar, A., Bawaneh, A., Awwad, M., Al-Hayek, H., Sijbrandij, M., Cuijpers, P., & Bryant, R. A. (2021). A longitudinal study of mental health before and during the COVID-19 pandemic in Syrian refugees. European Journal of Psychotraumatology, 12(1), 1,991,651. https://doi.org/10.1080/20008198.2021.1991651	No depressive disorder, wrong focus	IC 1.2, 2.2
Baird, Martha; Bimali, Milan; Cott, Ashley; Brimacombe, Michael; Ruhland-Petty, Therese; Daley, Christine (2017): Methodological challenges in conducting research with refugee women. In: Issues Ment Health Nurs 38 (4), S. 344–351. https://doi.org/10.1080/01612840.2017.1291775	No depressive disorder	IC 1.2
Bastin, P.; Bastard, M.; Rossel, L.; Melgar, P.; Jones, A.; Antierens, A. (2013): Description and predictive factors of individual outcomes in a refugee camp based mental health intervention (Beirut, Lebanon). In: PLoS ONE [Electronic Resource] 8 (1), e54107. https://doi.org/10.1371/journal.pone.0054107	Internally displaced persons	EC 1
Blackwell, M. A., Yeater, E. A., Ahmad, S., & Selmo, P. (2022). Sustaining psychotherapist effectiveness and independence: An exploratory study with displaced persons in Kurdistan, Iraq. Traumatology. Advance online publication. https://doi.org/10.1037/trm0000417	No depressive disorder	IC 1.2
Boege, K., Karnouk, C [C.], Hoell, A [A.], Tschorn, M., Kamp-Becker, I [I.], Padberg, F [F.], Ubleis, A., Hasan, A [A.], Falkai, P [P.], Salize, H. J., Meyer-Lindenberg, A., Banaschewski, T [T.], Schneider, F., Habel, U [U.], Plener, P [P.], Hahn, E [E.], Wiechers, M [M.], Strupf, M [M.], Jobst, A.,... Bajbouj, M [M.] (2022). Effectiveness and cost-effectiveness for the treatment of depressive symptoms in refugees and asylum seekers: A multi-centred randomized controlled trial. LANCET REGIONAL HEALTH-EUROPE, 19. https://doi.org/10.1016/j.lanepe.2022.100413	Age < 18 included	IC 1.1
Bryant, R. A., Bawaneh, A., Awwad, M., Al-Hayek, H., Giardinelli, L., Whitney, C., Jordans, M. J. D., Cuijpers, P., Sijbrandij, M., Ventevogel, P., Dawson, K., Akhtar, A., & STRENGTHS Consortium (2022). Twelve-month follow-up of a randomised clinical trial of a brief group psychological intervention for common mental disorders in Syrian refugees in Jordan. Epidemiology and Psychiatric Sciences, 31, e81. https://doi.org/10.1017/S2045796022000658	No depressive disorder	IC 1.2
Buhmann, C. B.; Nordentoft, M.; Ekstroem, M.; Carlsson, J.; Mortensen, E. L. (2018): Long-term treatment effect of trauma-affected refugees with flexible cognitive behavioural therapy and antidepressants. In: Psychiatry Research 264, S. 217–223. https://doi.org/10.1016/j.psychres.2018.03.069	Primary focus on PTSD or trauma exposition	EC 2
Buhmann, C. B.C.; Nordentoft, M.; Ekstroem, M.; Carlsson, J.; Mortensen, E. L. (2016): The effect of flexible cognitive-behavioural therapy and medical treatment, including antidepressants on post-traumatic stress disorder and depression in traumatised refugees: pragmatic randomised controlled clinical trial. In: British Journal of Psychiatry 208 (3), 252- + . https://doi.org/10.1192/bjp.bp.114.150961	Primary focus on PTSD or trauma exposition	EC 2
Buhmann, C.; Mortensen, E. L.; Nordentoft, M.; Ryberg, J.; Ekstrom, M. (2015): Follow-up study of the treatment outcomes at a psychiatric trauma clinic for refugees. In: Torture 25 (1), S. 1–16. Online verfügbar unter https://ovidsp.ovid.com/ovidweb.cgi?T=JS&CSC=Y&NEWS=N&PAGE=fulltext&D=med12&AN=26021344	Primary focus on PTSD or trauma exposition	EC 2
Carlsson, Jessica Mariana; Mortensen, Erik Lykke; Kastrup, Marianne (2005): A Follow-Up Study of Mental Health and Health-Related Quality of Life in Tortured Refugees in Multidisciplinary Treatment. In: J Nerv Ment Dis 193 (10), S. 651–657. https://doi.org/10.1097/01.nmd.0000180739.79884.10	No depressive disorder	IC 1.2
Carlsson, Jessica Mariana; Olsen, Dorte Reff; Kastrup, Marianne; Mortensen, Erik Lykke (2010): Late mental health changes in tortured refugees in multidisciplinary treatment. In: J Nerv Ment Dis 198 (11), S. 824–828. https://doi.org/10.1097/NMD.0b013e3181f97be3	No depressive disorder	IC 1.2
Carlsson, Jessica Mariana; Olsen, Dorte Reff; Mortensen, Erik Lykke; Kastrup, Marianne (2006): Mental Health and Health-Related Quality of Life: A 10-Year Follow-Up of Tortured Refugees. In: J Nerv Ment Dis 194 (10), S. 725–731. https://doi.org/10.1097/01.nmd.0000243079.52138.b7	Not an intervention study	IC 6
Corna, F.; Tolail, F.; Chowdhury, M. R.R.; Bizouerne, C. (2019): Supporting maternal mental health of Rohingya refugee women during the perinatal period to promote child health and wellbeing: a field study in Cox's Bazar. In: INTERVENTION-INTERNATIONAL JOURNAL OF MENTAL HEALTH PSYCHOSOCIAL WORK AND COUNSELLING IN AREAS OF ARMED CONFLICT 17 (2), S. 160–168. https://doi.org/10.4103/INTV.INTV_28_19	Age < 18 and Depression diagnosis not met	IC 1.1; 1.2
Danner, C. C.; Robinson, B. B.E.; Striepe, M. I.; Rhodes, P. F.Y. (2007): Running from the demon: Culturally specific group therapy for depressed Hmong women in a family medicine residency clinic. In: WOMEN & THERAPY 30 (1–2), S. 151–176. https://doi.org/10.1300/J015v30n01_07	Not only refugees and asylum seekers	IC 1.3
Davies, J.; McKenna, M.; Bayley, J.; Denner, K.; Young, H. (2020): Using engagement in sustainable construction to improve mental health and social connection in disadvantaged and hard to reach groups: a new green care approach. In: Journal of Mental Health 29 (3), S. 350–357. https://doi.org/10.1080/09638237.2020.1714001	Not only refugees and asylum seekers	IC 1.3
de Am Graaff; Cuijpers, P.; McDaid, D.; Park, A.; Woodward, A.; Bryant, R. A. et al. (2020): Peer-provided Problem Management Plus (PM plus) for adult Syrian refugees: a pilot randomised controlled trial on effectiveness and cost-effectiveness. In: EPIDEMIOLOGY AND PSYCHIATRIC SCIENCES 29. https://doi.org/10.1017/S2045796020000724	No depressive disorder	IC 1.2
Dix-Peek, D.; Werbeloff, M. (2018): Evaluation of the efficacy of a South African psychosocial model for the rehabilitation of torture survivors. In: Torture 28 (1), S. 34–57. Online verfügbar unter https://ovidsp.ovid.com/ovidweb.cgi?T=JS&CSC=Y&NEWS=N&PAGE=fulltext&D=med15&AN=30047490	Not only refugees and asylum seekers	IC 1.3
Dzubur-Kulenovic, A.; Weine, S. M.; Pavkovic, I. (2001): [Psychotherapeutic testimony be refugees from Bosnia-Herzegovina: a pilot study]. [Croatian]. In: Medicinski Arhiv 55 (1 Suppl 1), S. 47–51. Online verfügbar unter https://ovidsp.ovid.com/ovidweb.cgi?T=JS&CSC=Y&NEWS=N&PAGE=fulltext&D=med4&AN=11795194	Primary focus on PTSD or trauma exposition	EC 2
ElBarazi, A. S., Tikamdas, R., Ahmed, S., & Ramadan, S. (2022). Cognitive Processing Therapy for the Treatment of PTSD, Depression and Anxiety in Syrian Refugees in Egypt. INTERVENTION-INTERNATIONAL JOURNAL of MENTAL HEALTH PSYCHOSOCIAL WORK and COUNSELLING in AREAS of ARMED CONFLICT, 20(2), 179–187. https://doi.org/10.4103/intv.intv_33_21	No depressive disorder	IC 1.2
ElBarazi, A. (2023). Cognitive Processing Therapy for the Treatment of PTSD, Depression, Anxiety Symptoms and Difficulties in Emotion Regulation in Syrian Refugee Women Exposed to Intimate Partner Violence. Intervention Journal of Mental Health and Psychosocial Support in Conflict Affected Areas, 21(2). https://journals.lww.com/invn/fulltext/2023/21020/cognitive_processing_therapy_for_the_treatment_of.3.aspx	No depressive disorder	IC 1.2
Fox, P. G.; Cowell, J. M.; Montgomery, A. C.; Willgerodt, M. A. (1998): Southeast Asian refugee women and depression: a nursing intervention. In: International Journal of Psychiatric Nursing Research 4 (1), S. 423–432. Online verfügbar unter https://ovidsp.ovid.com/ovidweb.cgi?T=JS&CSC=Y&NEWS=N&PAGE=fulltext&D=med4&AN=10474398	Full-text not available	EC 5.3
Fox, Raymond (1984): The Indochinese: Strategies for health survival. In: Int J Soc Psychiatry 30 (4), S. 285–291. https://doi.org/10.1177/002076408403000405	No intervention study	IC 6
Goodkind, Jessica R.; Bybee, Deborah; Hess, Julia Meredith; Amer, Suha; Ndayisenga, Martin; Greene, R. Neil et al. (2020): Randomized controlled trial of a multilevel intervention to address social determinants of refugee mental health. In: Am J Community Psychol 65 (3–4), S. 272–289. https://doi.org/10.1002/ajcp.12418	No depressive disorder	IC 1.2
Graaff, Anne M. de; Cuijpers, Pim; Acarturk, Ceren; Bryant, Richard; Burchert, Sebastian; Fuhr, Daniela C. et al. (2020): Effectiveness of a peer-refugee delivered psychological intervention to reduce psychological distress among adult Syrian refugees in the Netherlands: Study protocol. In: Eur J Psychotraumatol 11 (1). https://doi.org/10.1080/20008198.2019.1694347	Study Protocol	IC 5
Greene, M. C., Muro, M., Kane, J. C., Young, E., Paniagua-Avila, A., Miller-Suchet, L.,... Verdeli, H. (2024). Task Sharing and Remote Delivery of Brief Interpersonal Counseling for Venezuelan Migrants and Refugees Living in Peru during the COVID-19 Pandemic: A Mixed-Methods Pilot Study. *International Journal of Environmental Research and Public Health*, *21*(2). https://doi.org/10.3390/ijerph21020166	Not only refugees and asylum seekers	IC 1.3
Haddad Kreidie, L., Sakhi, S., Wardani, F., AlSabah, I., & Anbar, K. The Impact of Dramatherapy on the Mental Health of Socioeconomically Disadvantaged and Refugee Women in Lebanon. *Journal of Social Service Research*, 1–17. https://doi.org/10.1080/01488376.2024.2349236	Not only refugees and asylum seekers	IC 1.3
Heim, E.; Ramia, J. A.; Hana, R. A.; Burchert, S.; Carswell, K.; Cornelisz, I. et al. (2021): Step-by-step: Feasibility randomised controlled trial of a mobile-based intervention for depression among populations affected by adversity in Lebanon. In: INTERNET INTERVENTIONS-THE APPLICATION OF INFORMATION TECHNOLOGY IN MENTAL AND BEHAVIOURAL HEALTH 24. https://doi.org/10.1016/j.invent.2021.100380	Not only refugees and asylum seekers	IC 1.3
Husby, Simon Ruben; Carlsson, Jessica; Mathilde Scotte Jensen, Anna; Glahder Lindberg, Laura; Sonne, Charlotte (2020): Prevention of trauma‐related mental health problems among refugees: A mixed‐methods evaluation of the mindspring group programme in denmark. In: J Community Psychol 48 (3), S. 1028–1039. https://doi.org/10.1002/jcop.22323	No depressive disorder	IC 1.2
Jeon, S.; Lee, J.; Jun, J. Y.; Park, Y. S.; Cho, J.; Choi, J. et al. (2020): The Effectiveness of Cognitive Behavioral Therapy on Depressive Symptoms in North Korean Refugees. In: PSYCHIATRY INVESTIGATION 17 (7), S. 681–687. 10.30773/pi.2019.0134	No depressive disorder	IC 1.2
Kalra, N., Habumugisha, L., & Shankar, A. (2024). Impacts of an abbreviated personal agency training with refugee women and their male partners on economic empowerment, gender-based violence, and mental health: A randomized controlled trial in Rwanda. *BMC Public Health*, *24*(1), 1306. 10.1186/s12889-024-18780-8	No depressive disorder	IC 1.2
Khoury, B., & Daouk, S. (2022). Problem-solving skills groups for female Syrian refugees in Lebanon: a study of a mental health intervention. JOURNAL of REFUGEE STUDIES, 35(1), 662–674. 10.1093/jrs/feab099	Internally displaced persons and others included	EC 1
Lee, M.‑S., Seo, Y. E., Mok, Y. E., & Lee, S. H. (2021). Heart Rate Variability after Treatment for Depression in North Korean Defectors. Applied Psychophysiology and Biofeedback, 46(1), 11–18. 10.1007/s10484-020-09491-y	No psychological intervention	IC 2.1
Lehnung, Maria; Shapiro, Elan; Schreiber, Melanie; Hofmann, Arne (2017): Evaluating the EMDR Group Traumatic Episode Protocol with refugees: A field study. In: JOURNAL OF EMDR PRACTICE AND RESEARCH 11 (3), S. 129–138. 10.1891/1933-3196.11.3.129	No depressive disorder	IC 1.2
Lindegaard, T., Seaton, F., Halaj, A., Berg, M., Kashoush, F., Barchini, R., Ludvigsson, M., Sarkohi, A., & Andersson, G. (2021). Internet-based cognitive behavioural therapy for depression and anxiety among Arabic-speaking individuals in Sweden: a pilot randomized controlled trial. Cognitive Behaviour Therapy, 50(1), 47–66. 10.1080/16506073.2020.1771414	Other migrants included	EC1
Maleku, A., Subedi, B., Kim, Y. K., Haran, H., & Pyakurel, S. (2024). Toward healing-centered engagement to address mental well-being among young Bhutanese-Nepali refugee women in the United States: Findings from the cultural leadership project. *Journal of Ethnic & Cultural Diversity in Social Work: Innovation in Theory, Research & Practice*, *33*(3), 167–185. 10.1080/15313204.2022.2161684	Age < 18	IC 1.1
Mateos-Fernandez, R., & Saavedra, J. (2022). Designing and assessing of an art-based intervention for undocumented migrants. Arts & Health, 14(2), 119–132. 10.1080/17533015.2020.1866623	No depressive disorder	IC 1.2
Medrano, S. G.; Panhofer, H. (2019): Contributions of Dance Movement Therapy`s on the improvement of life and health of migrants. C. In: REVISTA INCLUSIONES 6, S. 97–116	No depressive disorder	IC 1.3
Meffert, Susan M.; Abdo, Akram Osman; Alla, Omayma Ahmed Abd; Elmakki, Yasir Omer Mustafa; Omer, Afrah Abdelrahim; Yousif, Sahar et al. (2014): A pilot randomized controlled trial of interpersonal psychotherapy for Sudanese refugees in Cairo, Egypt. In: Psychol Trauma 6 (3), S. 240–249. 10.1037/a0023540	No depressive disorder	IC1.2
Mitschke, Diane B.; Aguirre, Regina T. P.; Sharma, Bonita (2013): Common threads: Improving the mental health of Bhutanese refugee women through shared learning. In: Soc Work Ment Health 11 (3), S. 249–266. 10.1080/15332985.2013.769926	No psychological intervention	IC 2.1
Mollica, Richard F.; Wyshak, Grace; Lavelle, James; Truong, Toan; Tor, Svang; Yang, Ten (1990): Assessing symptom change in Southeast Asian refugee survivors of mass violence and torture. In: Am J Psychiatry 147 (1), S. 83–88. 10.1176/ajp.147.1.83	No depressive disorder	IC 1.2
Nordbrandt, M. S., Vindbjerg, E., Mortensen, E. L [Erik Lykke], & Carlsson, J [Jessica] (2022). Chronicity of posttraumatic stress disorder and comorbid pain as predictors of treatment response for trauma-affected refugees. Journal of Traumatic Stress, 35(5), 1393–1404. 10.1002/jts.22839	Age < 18	IC 1.1
Opaas, M., Wentzel-Larsen, T., & Varvin, S. (2022). Predictors of the 10 year course of mental health and quality of life for trauma-affected refugees after psychological treatment. EUROPEAN JOURNAL of PSYCHOTRAUMATOLOGY, 13(1). 10.1080/20008198.2022.2068910	No depressive disorder	IC 1.2
Orang, M., Missmahl, I., Gardisi, M., & Kluge, U. Internet-Delivered Value Based Counseling (VBC) Aimed at the Reduction of Post-Migration Psychosocial Stress-A Pilot Study. JOURNAL of TECHNOLOGY in HUMAN SERVICES. Advance online publication. 10.1080/15228835.2022.2156973	Other migrants and residents included	EC 1
Orang, T. M., Missmahl, I., am Thoele, Valensise, L., Brenner, A., Gardisi, M., Peter, H., & Kluge, U. (2022). New directions in the mental health care of migrants, including refugees-A randomized controlled trial investigating the efficacy of value-based counselling. CLINICAL PSYCHOLOGY & PSYCHOTHERAPY, 29(4), 1433–1446. 10.1002/cpp.2728	Migrants included	EC 1
Poudel-Tandukar, K., Jacelon, C. S., Poudel, K. C., Bertone-Johnson, E. R., Rai, S., Ramdam, P., & Hollon, S. D. (2022). Mental health promotion among resettled Bhutanese adults in Massachusetts: Results of a peer-led family-centred Social and Emotional Well-being (SEW) intervention study. HEALTH & SOCIAL CARE in the COMMUNITY, 30(5), 1869–1880. 10.1111/hsc.13566	Depression not focus of the intervention	IC 2.2
Poudel-Tandukar, K., Jacelon, C. S., Rai, S., Ramdam, P., Bertone-Johnson, E. R., & Hollon, S. D. (2021). Social and Emotional Wellbeing (SEW) Intervention for Mental Health Promotion Among Resettled Bhutanese Adults in Massachusetts. COMMUNITY MENTAL HEALTH JOURNAL, 57(7), 1318–1327. 10.1007/s10597-020-00754-w	Depression is not focus of the intervention	IC 2.2
Rafla, J., Schwartz, K., Yoshikawa, H., Hilgendorf, D., Ramachandran, A., Khanji, M.,... Wuermli, A. (2024). Cluster randomized controlled trial of a phone-based caregiver support and parenting program for Syrian and Jordanian families with young children. *Early Childhood Research Quarterly*, *69*, 141–153. 10.1016/j.ecresq.2024.07.004	No depressive disorder	IC 1.2
Raghavan, Sumithra; Rasmussen, Andrew; Rosenfeld, Barry; Keller, Allen S. (2013): Correlates of symptom reduction in treatment-seeking survivors of torture. In: Psychol Trauma 5 (4), S. 377–383. 10.1037/a0028118	No depressive disorder	IC 1.2
Rawlinson, R.; Aslam, R. W.; Burnside, G.; Chiumento, A.; Eriksson-Lee, M.; Humphreys, A. et al. (2020): Lay-therapist-delivered, low-intensity, psychosocial intervention for refugees and asylum seekers (PROSPER): protocol for a pilot randomised controlled trial. In: TRIALS 21 (1). 10.1186/s13063-020-04310-5	Study Protocol	IC 5
Renner, Walter; Bänninger-Huber, Eva; Peltzer, Karl (2011): Culture-Sensitive and Resource Oriented Peer (CROP)-Groups as a community based intervention for trauma survivors: A randomized controlled pilot study with refugees and asylum seekers from Chechnya. In: Australasian Journal of Disaster and Trauma Studies 2011 (1), S. 1–13. https://doi.org/10.1037/t07469-000	No depressive disorder	IC 1.2
Robertson, Cheryl L.; Halcon, Linda; Hoffman, Sarah J.; Osman, Nadifa; Mohamed, Amin; Areba, Eunice et al. (2019): Health realization community coping intervention for Somali refugee women. In: J Immigr Minor Health 21 (5), S. 1077–1084. 10.1007/s10903-018-0804-8	No depressive disorder	IC 1.2
Sandahl, H., Carlsson, J [J.], Sonne, C [C.], Mortensen, E. L [E. L.], Jennum, P., & Baandrup, L. (2021). Investigating the link between subjective sleep quality, symptoms of PTSD, and level of functioning in a sample of trauma-affected refugees. SLEEP, 44(9). 10.1093/sleep/zsab063	No depressive disorder neither focus, Predictor analysis	IC 1.2, 2.2, 6
Sardana, S. Coping with depression: A dynamic networks approach to the study of social network constellation, cohesion and conflict (2-B) [, ProQuest Information & Learning]. RIS. https://search.ebscohost.com/login.aspx?direct=true&db=psyh&AN=2023-01886-271&lang=de&site=ehost-live	No original study	EC 5.1
Slewa-Younan, Shameran; McKenzie, Molly; Thomson, Russell; Smith, Mitchell; Mohammad, Yaser; Mond, Jonathan (2020): Improving the mental wellbeing of Arabic speaking refugees: An evaluation of a mental health promotion program. In: BMC Psychiatry 20. 10.1186/s12888-020-02732-8	No depressive disorder	IC 1.2
Small, Eusebius; Kim, Youn Kyoung; Praetorius, Regina T.; Mitschke, Diane B. (2016): Mental health treatment for resettled refugees: A comparison of three approaches. In: Soc Work Ment Health 14 (4), S. 342–359. https://doi.org/10.1080/15332985.2015.1080205	No depressive disorder	IC 1.2
Sonne, C [Charlotte], Mortensen, E. L [Erik Lykke], Silove, D [Derrick], Palic, S., & Carlsson, J [Jessica] (2021). Predictors of treatment outcomes for trauma-affected refugees—results from two randomised trials. Journal of Affective Disorders, 282, 194–202. https://doi.org/10.1016/j.jad.2020.12.095 Strupf, M [Michael], Wiechers, M [Maren], Bajbouj, M [Malek], Böge, K., Karnouk, C [Carine], Goerigk, S., Kamp-Becker, I [Inge], Banaschewski, T [Tobias], Rapp, M [Michael], Hasan, A [Alkomiet], Falkai, P [Peter], Jobst-Heel, A., Habel, U [Ute], Stamm, T., Heinz, A [Andreas], Hoell, A [Andreas], Burger, M., Bunse, T., Hoehne, E [Edgar],... Padberg, F [Frank] (2023). Predicting treatment outcomes of the empowerment group intervention for refugees with affective disorders: Findings from the MEHIRA project. Journal of Affective Disorders, 323, 241–250. https://doi.org/10.1016/j.jad.2022.11.050	Predictor analysis	IC 6
Tay, A. K., Mohsin, M., Foo, C. Y., Rees, S., & Silove, D [D.]. Long-term efficacy of brief psychological treatments for common mental disorders in Myanmar refugees in Malaysia: 12-month follow-up of a randomized, active-controlled trial of integrative adapt therapy v. cognitive behavioral therapy. PSYCHOLOGICAL MEDICINE. Advance online publication. https://doi.org/10.1017/S0033291722003245	Same sample	EC 5.2
Tol, W. A.; Augustinavicius, J.; Carswell, K.; Brown, F. L.; Adaku, A.; Leku et al. (2018): Translation, adaptation, and pilot of a guided self-help intervention to reduce psychological distress in South Sudanese refugees in Uganda. In: GLOBAL MENTAL HEALTH 5. https://doi.org/10.1017/gmh.2018.14	No depressive disorder	IC 1.2
Tol, W. A.; Leku; Lakin, D. P.; Carswell, K.; Augustinavicius, J.; Adaku, A. et al. (2020): Guided self-help to reduce psychological distress in South Sudanese female refugees in Uganda: a cluster randomised trial. In: LANCET GLOBAL HEALTH 8 (2), E254-E263	No depressive disorder	IC 1.2
Tran, Sophia N.: Short term, time limited cognitive behavioral therapy of major depression with Vietnamese refugee women: An analysis of three cases. ProQuest Information & Learning. Online verfügbar unter https://search.ebscohost.com/login.aspx?direct=true&db=psyh&AN=2006–99002-123&lang=de&site=ehost-live	Case Study	IC 6
Uldall, S. W., Poulsen, D. V., Christensen, S. I., Wilson, L., & Carlsson, J [J.] (2022). Mixing Job Training with Nature-Based Therapy Shows Promise for Increasing Labor Market Affiliation among Newly Arrived Refugees: Results from a Danish Case Series Study. INTERNATIONAL JOURNAL of ENVIRONMENTAL RESEARCH and PUBLIC HEALTH, 19(8). 10.3390/ijerph19084850	No full text available	EC 5.3
van Es, C. M., Boelen, P. A., Zwaanswijk, M., Te Brake, H., & Mooren, T. (2021). Family Empowerment (FAME): A feasibility trial of preventive multifamily groups for asylum seeker families in the Netherlands. Journal of Marital and Family Therapy, 47(4), 864–881. 10.1111/jmft.12539	Age < 18 and depression not focus	IC 1.1, 2.2
Weine, Stevan M.; Kulenovic, Alma Dzubur; Pavkovic, Ivan; Gibbons, Robert (1998): Testimony psychotherapy in Bosnian refugees: A pilot study. In: Am J Psychiatry 155 (12), S. 1720–1726. 10.1176/ajp.155.12.1720	No depressive disorder	IC 1.2
Weinstein, Netta; Khabbaz, Farah; Legate, Nicole (2016): Enhancing need satisfaction to reduce psychological distress in Syrian refugees. In: J Consult Clin Psychol 84 (7), S. 645–650. 10.1037/ccp0000095	No depressive disorder	IC 1.2
Westermeyer, Joseph (1988): A matched pairs study of depression among Hmong refugees with particular reference to predisposing factors and treatment outcome. In: Soc Psychiatry Psychiatr Epidemiol 23 (1), S. 64–71. 10.1007/BF01788445	< 18 years	IC 1.1

### Populations investigated and background of investigators

Twenty studies fulfilled the inclusion criteria. Apart from Mateos-Fernándes et al. [31], which treated patients from the Sub Sahara region (Africa) and Poudel-Tandoukar et al. [32] investigating a Ukrainian refugee population (Europe), the patients in the remaining trials were all from Asia: five studies with refugees from Burma (Myanmar), four studies with refugees from Afghanistan/Iran, four with refugees from Syria, one mixed with Afghanistan/Iran and Syria, one study each with refugees from Sri Lanka, Cambodia and Bhutan. Seventeen interventions were culturally adaptions of an already existing therapy program or especially developed for refugees [32–39], the rest of the publications did not provide enough information in this regard. Apart from two first authors, the Indian author Vijayakumar and the Turkish author Acarturk, all first authors were from high income countries (6 from the USA, 5 from Germany, 2 from Netherlands, 4 from Australian, 1 from Spain). The host countries of the refugees were mainly high-income countries, in particular the USA and Europe (k = 10 studies). Three studies were conducted in Middle Income Countries [34, 40, 41] and two [33, 36] in low income countries (see Tables 4 and 5).

**Table 4:** Psychological interventions of the studies included (k = 20)

First author (year) [country of first author]	Name of treatment	Number of sessions/ Dose	Countries/ regions of origin or ethnicity (host country)	Interpreter used/culturally tailored intervention	Therapists	Cultural adaptation process
Quantitative randomized controlled trials						
Acarturk (2024) [Turkey] [38]	Group problem Management plus (gPM +)	5, weekly	Syrian (Turkey)	no/yes	(non-specialist) facilitators	Adaption on language, metaphors and contextual factors: rapid qualitative assessments (free listing interviews, key informant interviews and focus group discussions) were conducted to understand important cultural and linguistic elements about Syrian culture. Adaptation recommendations based on rapid qualitative assessment were presented to key stakeholders including local and international healthcare providers, academics and policymakers
Bolton (2014) [USA] [40]	Common Elements Treatment Approach (CETA)	average of 9.7 weekly	Myanmare, Others (Thailand)	no/yes	Lay workers (refugees)	A research and program development model, DIME (Design, Implementation, Monitoring and Evaluation) was used to select and adapt the psychosocial assessment tools and therapeutic treatments. Successful adaptations were: (1) fewer items, (2) simplified language, (3) brief step-by-step instructions for each item (4) complex concepts of cognitive coping simplified (5) training of laypersons
Cuijpers (2022) [Netherlands] [36]	Step-by-Step	5 digital sessions (one session per week, 20 min), so all in all 5–8 weeks	Syrian (Lebanon)	no/yes	Coordinator and nonspecialists (e-helpers) with background in psychology or health	The adaptation process is not described. Outlined result of the adaptation: Participants can choose the appearance of the character, broadly reflecting the main cultural groups in Lebanon (Lebanese, Syrians, and Palestinians)
De Graaff (2023) [Netherlands] [35]	Problem Management Plus (PM +)	5 in person or video, weekly (90 min)	Syrian (Netherlands)	no/yes	Lay workers (Syrian refugees)	Described process of adaptation: (1) literature review, (2) stakeholder engagement, (3) rapid qualitative assessments, (4) literal translation, (5) cognitive interviews, (6) adaptation workshops, (7) finalization of manuals
Kananian (2020) [Germany] [46]	Culturally adapted CBT plus problem solving (CA-CBT +)	12 in 6 weeks	Afghan/Iranian (Germany)	no/yes	Health professionals (Farsi-speaking)	NA
Northwood (2020) [USA] [43]	Intensive psychotherapy and case management (IPCM)	average 41.3 psychotherapy sessions and 38.3 case management sessions; 1 year treatment, weekly or bi-weekly;	Karen refugees from Myanmar(USA)	yes, if needed/yes	Health professionals (Psychotherapists and social workers)	NA
Tay (2020) [Australia] [34]	Integrative Adapt Therapy (IAT)	6, weekly (45 min)	Myanmare (Malaysia)	no/yes	Lay counsellors under supervision	After translation, an iterative qualitative research process refined translation accuracy and cultural relevance. Feedback was gathered from focus groups and key informants from three ethnic groups. Culturally relevant metaphors and idioms were incorporated to enhance understanding
Wiechers (2023) [Germany] [39]	Empowerment group intervention (Empowerment)	2/week in the first 4 weeks; 1/week in the last 8 weeks (90 min)	Syrian/Iraqi (Germany)	yes/yes	Health professionals (Psychologists)	The adaptation process was not described. Outlined results of the adjustments: Teaching of opportunities for behavioral activation and sleep hygiene in mass shelters; Inclusion of religion and cultural values, such as family cohesion; culturally sensitive group compositions for participants; utilization of linguistic and cultural mediators to enhance engagement and effectiveness
Vijayakumar (2017) [India] [41]	Contact and Safety Planning (CASP)	biweekly for 6 months	Sri Lankan (India)	no/NA	Lay counsellors (community volunteers/refugees)	NA
Berkson (2014) [USA] [49]	Cambodian Health Promotion Program (CHPP)	5	Cambodian (USA)	yes/yes	Heath professional and a bicultural health educator	The adaptation process was not described. Outlined results of the adjustments: co-facilitate sessions with an American psychologist and a Cambodian health worker. The curriculum is kept simple, using resources in Khmer to suit the language level of the participants
Brakemeier (2017) [Germany] [45]	Interpersonal Integrative Therapy for refugees (IITF)	10 psychotherapy (each 100 min); 4 social worker talk (each 50 min); 1 occupational therapy week (optional); 3 psychiatric treatments (optional)	Syrian/Iraqi (Germany)	yes/yes	Health professionals: Psychological and medical psychotherapists (in training), social workers, occupational therapists	NA
Kananian (2017) [Germany] [47]	Culturally Adapted CBT (CA-CBT)	12, weekly (each 90 min)	Afghan/Iranian (Germany)	no/yes	Health professionals (Farsi-speaking)	Conducting a one-hour open-ended interview to collect the patients' individual perspectives on their mental health problems. Discussion of the results in the working group (no systematic evaluation with qualitative methods) as well as general aspects of the culture of Farsi-speaking refugees, which were to be incorporated into the manual. The adaptations of the manual to the Afghan culture included four dimensions: (1) perception and assessment of symptoms, (2) explanation of causes, (3) application of local therapeutic practices and concepts. Also including examples and metaphors from the Afghan culture and the number of sessions was reduced to 12
Kinzie (2012) [USA] [42]	Torture Treatment Program	average 14; 1-year treatment;	Iranian, Afghan, Ethiopian, Somali (USA)	yes/NA	Health professionals (Psychiatrists, Counsellors)	NA
Mateos-Fernandez (2020) [Spain] [31]	Art-based intervention (ABI)	6 in two months	Sub-Saharan-African (Spain)	yes/yes	Health professionals	The adaptation process was not fully described. The second author supervised the intervention's development, focusing on reducing linguistic and cultural barriers. Activities were designed to be simple to minimize potential barriers. Original music and songs from participants' native countries were used as content to help bridge cultural gaps
Pishyar(2012) [Australia] [44]	De-reflection intervention/ Logo therapy	12, weekly	Afghani (Australia)	NA	Health professionals (Psychologists)	NA
Poudel-Tandukar (2021) [USA] [53]	Social and Emotional Wellbeing (SEW)	5, once weekly	Bhutanese (USA)	NA/yes	Health professionals	A series of community meetings. Workshops were conducted using a six-step intervention mapping tool which included: (1) analyzing problems, (2) setting priorities, (3) setting objectives, (4) developing program components, (5) identifying evidence-based programs (6) developing a program implementation and evaluation plan
Poudel-Tandukar (2024) [USA] [32]	Social and Emotional Wellbeing (SEW)	5, weekly	Ukrainian (USA)	yes/yes	Health professionals	The intervention session was developed in collaboration with community partners, holistic nurses, and psychological experts. Community members were involved in the design process, from conception to implementation to evaluation
Stammel (2017) [Germany] [48]	Multidisciplinary treatment for traumatized refugees in a naturalistic setting	once or twice a week, average of 14 months	14 different countries/regions (Germany)	yes/yes	Health professionals (psychotherapists (behavioral, psychodynamic, systemic), social workers; psychiatrists)	NA
Tay (2021) [Australia] [33]	Group Integrative Adapt Therapy (IAT-G)	7 sessions group-intervention weekly (90 min)	Rohingyan (Bangladesh)	no/yes	Lay counsellors (IAT trainees who were already providing services in Cox’s Bazar)	NA
Van Wyk (2012) [Australia] [50]	Standard intervention provided by a resettlement organisation	average of 11 service contacts (4 assessment sessions, 6–7 therapy sessions; range 0–33) over 7 months	Myanmare (Australia)	yes/yes	Health professionals (n = 4 psychologists, n = 1 social worker, n = 1 counsellor)	NA

**Table 5:** Study design data in the studies included (k = 20)

First author (year)	Study design [control / comparison]	Age, mean (SD), range (in years)	Sample size: in pre-post analyses (% female, pre)	Dropout (%)	Allowed Medication	Allowed parallel Psychotherapy or TAU	Funding
Quantitative randomized controlled trials							
Acarturk (2024) [38]	RCT **[CAU]**	37.15 (11.21), 18 +	368 (69.6); 143 subgroup probably depressed	10%	NA	NA	Horizon 2020 Framework Programme for Research and Innovation
Bolton (2014) [40]	RCT [waitlist]	36.5 (12.6),18–85	347 (63)	21%	NA	No	US Agency for International Developement (USAID) Victims of Torture Fund
Cuijpers (2022) [36]	RCT (randomized clinical trial) [enhanced care as usual (ECAU)]	31.5 (8.7), NA IG: 31.4 (8.5)/ CG: 31.6 (8.9)	569 (58.3)	46.2% post; 52,7% follow up	NA	NA	Research for Health in Humanitarian Crisis (R2HC, managed by Erlha)
De Graaff (2023) [35]	RCT [CAU]	36.52 (11.72), 18–69 IG: 36.35 (11.97), 18–69/ CG: 36.69 (11.52), 19–67	Pre: 206 (38.3%) Post: 178	Assessed: 236 Randomized: 206 Post: 178 Follow-up: 148 Randomized/Post: 13.59%	Yes	Yes, but no treatment in specialized mental health care in treatment group	European Union’s Horizon 2020 research and Innovation Programme Societal Challenges under grant agreement
Kananian (2020) [46]	RCT [waitlist]	22.1 (3.6), 18–29	24 (-)	4%	No	Yes	Sponsors of the Johann Wolfgang Goethe University; Foundation Polytechnic Society Frankfurt am Main
Northwood (2020) [43]	GRT [care as usual from primary care provider]	42.8 (3.3), NA	222 (80)	10%	Yes	No	Diverse foundations and the State of Minnesota
Tay (2020) [34]	RCT [Cognitive Behavioral Therapy (CBT)]	30.8 (9.6), 18–70	322 (28.1)	3%	NA	No	National Health and Medical Research Council Australia
Wiechers (2023) [39]	RCT [Empowerment intervention/TAU]	ITT Intervention: 32.62 (9.08); ITT TAU: 31.64 (9.84); PP Intervention: 31.87 (8.98); PP TAU: 32.57 (10.80); 18–65	149 (Intervention 43.2; TAU 32.4)	47%	NA	Yes	Innovationsfond and the German Ministry of Health
Vijayakumar (2017) [41]	GRT [Control Camp]	IG: 41.6 (15.0) / CG: 39.1 (15.1)	suicidality: 1.755 (61% Intervention; 56% Control); depression: 139 (58)	suicidality: NA depression: NA (52% persons did not receive intervention after recommendation)	No	Yes	ADRA and Sneha
Quantitative non-randomized trials							
Berkson (2014) [49]	Pre-post [none], Pilot study	NA (< 51: 22%, 51–60: 46%, > 60: 31%)	126 (64)	–	NA	NA	Dr. Albert S. Kuperman and the Albert Einstein College of medicine supported fellowship;
Brakemeier (2017) [45]	Pre-post [none]	34.1 (11.3 (18–60)	27 (24,3)	24%	Yes	No	Federal Ministry of Labor and Social Affairs (BMAS)
Kananian (2017) [47]	Pre-post [none]; Pilot study	25.6 (9.0), NA	7 (0)	22%	NA	No	Sponsors of the Johann Wolfgang Goethe University; Foundation Polytechnic Society Frankfurt am Main
Kinzie (2012) [42]	Pre-post [none]	48.0 (NA), 19–76	22 (59)	17%	Yes	No	–
Mateos-Fernandez (2020) [31]	Pre-post [none]; Pilot study	21.9 (NA),18–24	11 (0)	–	NA	NA	Spanish Government
Pishyar (2012) [44]	Pre-post [none]	38.5 (7.3), NA	16 (63)	–	No	NA	NA
Poudel-Tandukar (2021) [53]	Pre-post [none], Pilot study	30.6 (11.5), NA	44 (68)	–	NA	NA	University of Massachusetts
Poudel-Tandukar (2024) [32]	Pre-post [none], Pilot study	48.48 (17.07), 18 +	31 (74.19)	–	NA	NA	Blue Cross Blue Shield of Massachusetts Foundation
Stammel (2017) [48]	Pre-post [none]	25.4 (10.6), NA	76 (38)	–	Yes	NA	NA
Tay (2021) [33]	Single armed, assessor-blind nonrandomized	37.4 (12.8), 18- > 50	144 (41.7%); 66 Completer	Baseline to Post: 16.67%; Baseline to Follow up: 54.2%	NA	NA	United Nations High Commissioner for Refugees; National Health and Medical Research Council Australia
Van Wyk (2012) [50]	Pre-post [none]	34.1 (14.1), 18–80	62 (57)	11%	NA	NA	Australian Research Council; National Cancer Institute Minority Institution

*IG* Intervention group, *CG* Control group, *NA* Not available, *RCT* Randomized controlled trial, *GRT* Parallel group-randomized trial

Only five trials had 100% of participants with clinical depression [36, 39, 42–44]. Northwood et al. [43], Wiechers et al. [39] and Cuijpers et al. [36] were quantitative parallel group-randomized trial studies, the others limited through a pre-post design not involving a control group. In ten studies, depression was a necessary inclusion criterion, but in six of these studies, depression was only one inclusion criterion alongside other mental disorders like PTSD [33, 34, 36, 40, 43–46]. Six studies used a diagnostic interview to identify depression [42, 43, 45, 47, 48], while others applied a cut-off score of a self-report measure to identify patients with potential clinical depression. Kinzie et al. [42] was the only study relying on a diagnosis by a health professional conducting a diagnostic interview based on DSM IV. For more details about the specificity of each intervention's focus on depression, see Table 6.

**Table 6:** Strength of focus on depression in the studies included (k = 20)

First author (year)	Reported focus of the intervention	Diagnosis of Depressive Disorder (DD)	Is DD an Inclusion criteria (%DD)?
Quantitative randomized controlled trials			
Acarturk (2024) [38]	Depression and anxiety, PTSD/functional impairment, self-identified problems (2nd)	Hopkins Symptom Checklist (HSCL-25), depression subscale; cut-off score of > 1.75	No (47.8)
Bolton (2014) [40]	Comorbid mental health and functioning	Hopkins Symptom Checklist 25 (HSCL-25), depression subscale	Yes and/or PTSD (NA)
Cuijpers (2022) [36]	Depression, impaired functioning at posttreatment	Patient Health Questionnaire-9 (PHQ-9)	Yes and functional impairment (100)
De Graaff (2023) [35]	Depression, anxiety, post-traumatic stress disorder (2nd)	Hopkins Symptom Checklist-25 (HSCL-25)	No (68.9)
Kananian (2020) [46]	PTSD and Depression	Mini International Neuropsychiatric Interview (MINI)	Yes and other diagnoses (79)
Northwood (2020) [43]	Depression and PTSD	Structured Clinical Interview for DSM -IV (SKID)	Yes (100)
Tay (2020) [34]	Mental Health	Refugee Mental Health Assessment Package (RMHAP) depression module	Yes, and others (NA)
Wiechers (2023) [39]	Stress management, emotion regulation strategies, psychoeducation	Patient Health Questionnaire-9 (PHQ-9); Montgomery–Åsberg Depression Rating Scale (MÅDRS)	Yes (100)
Vijayakumar (2017) [41]	Suicidal behaviour (Depression)	Centre for epidemiological Studies-Revised (CESD-R)	No (24.7 intervention; 16.6 control)
Quantitative non-randomized trials			
Berkson (2014) [49]	Consequences of torture	Hopkins Symptom Checklist Cambodian Version (HSCL): scores greater than or equal to 1.75	No (52.8)
Brakemeier (2017) [45]	Mental Health	Structured Clinical Interview for DSM -IV (SKID)	Yes and other diagnoses (54)
Kananian (2017) [47]	Trauma related disorders	Mini International Neuropsychiatric Interview (MINI)	Yes and other diagnoses (56)
Kinzie (2012) [42]	Depression and PTSD	By psychiatrists after diagnostic interview based on DSM IV	No (100);
Mateos-Fernandez (2020) [31]	Depression and Anxiety	Beck`s Depression Inventory (BDI)	No (81)
Pishyar (2012) [44]	Depression	Depression Anxiety Stress Scale (DASS); Score > 28 = extremely severe depressed	Yes (100)
Poudel-Tandukar (2021) [53]	Stress, Anxiety and Depression	Hopkins Symptom Checklist (HSCL-25); Nepal Version; Scores > 1.75	No (48)
Poudel-Tandukar (2024) [32]	Stress, Anxiety and Depression; coping, self-afficacy, social support, and conflict resolution	Hopkins Symptom Checklist (HSCL-25), depression subscale; Mean Sore of > 2.1	No (61)
Stammel (2017) [48]	Trauma-related disorders (PTSD, Depression, Anxiety and Somatization)	Mini International Neuropsychiatric Interview (MINI)	No (86)
Tay (2021) [33]	Depression, posttraumatic stress disorder (PTSD), anxiety, functional impairment	Patient Health Questionnaire-9 (PHQ-9)	Yes and/or PTSD and functional impairment (NA)
Van Wyk (2012) [50]	Mental Health (PTSD, Depression, Anxiety and Somatization)	Hopkins Symptom Checklist-37 (HSCL-37)	No (37)

*DD* Depressive Disorder

### Effectiveness of included trials by study design and treatment format

The content and dose intensity of the interventions varied. The intervention dose ranged from 5 [36, 37, 49] to > 40 sessions [41, 43, 48]. The highest number of sessions was reported in trials using multidisciplinary treatments, which were non-manualized and in a naturalistic setting. Seventeen interventions were manualized. CBT was the most studied intervention with four trials [34, 40, 46, 47]. Nine interventions were performed in an individual and ten in a group setting, with one being delivered digitally[36]. Treatment was mainly carried out by health professionals, but the Common Elements Treatment Approach [40], the Integrative Adapt Therapy [33, 34], the Contact and Safety Planning (CSAP) [41] and the interventions developed by the World Health Organisation (WHO Problem Management Plus (PM +) and Step by Step [35, 36, 38] were carried out by lay counsellors under supervision. In nine trials, the use of an interpreter was reported [31, 39, 41–43, 45, 48–50].

### Studies involving a control condition

Nine studies used a control condition [34–36, 38–41, 43, 46]. Four were conducted in an individual setting, four in a group setting and one digitally. The sample sizes ranged from n = 24 [46] to n = 1755 [41], with the median of n = 322 participants.

#### Individual setting

Bolton et al. [40] examined the relative efficacy of a Common Elements Treatment Approach (CETA) compared to a waiting condition. CETA is a transdiagnostic treatment based on CBT, designed especially for delivery by non-professional providers in low-resource settings with few mental health professionals. It has a set of nine cross-cutting treatment components with decision-making rules and guidelines.

Problem Management Plus (PM +) was developed by the WHO as an intervention for underserviced communities and to be delivered by lay workers. PM + comprises 5 sessions: psychoeducation and stress management using diaphragmatic breathing (session 1), problem solving (session 2), behavioural activation by re-engaging with pleasant/task-oriented activities (session 3), accessing social support (session 4) and relapse prevention (session 5) [35]. De Graaf [35] compared PM + with a control group receiving care as usual which included all (mental) health services in the Netherlands. Additionally, PM + was delivered in a group setting in a trial by Acarturk et al. [38].

Northwood et al.[43] investigated the efficacy of the intensive psychotherapy and case management (IPCM) compared to a control group that received care as usual (CAU). CAU patients could be referred to a full range of behavioral health services by their primary care physician. IPCM is a behavioral health intervention consisting of psychotherapy and case management provided by refugee trauma specialists from the Centre for Victims of Torture (CVT) within two urban primary care clinics. The interventions were individually tailored and not manualized.

Vijayakumar et al. [41] compared two refugee camps in India, with one of the camps representing the passive control condition to the experimental condition called Contact and Safety Planning (CASP). The aim of the trial was to detect and reduce suicidal behavior among refugees, with depression representing a secondary focus. After a screening of all refugees in the camps, refugees with a high risk of committing suicide were observed by community volunteers who used safety planning cards. The community volunteers made periodic visits to provide emotional support to individuals who were depressed or suicidal. The safety planning card consisted of an individualized list of coping strategies containing names and contact numbers of persons in the individual`s immediate family, social circle and health services who could be contacted in a crisis [41].

#### Group setting

Kananian [46] compared Culturally Adapted Cognitive Behavioural Therapy plus Problem Solving (CA-CBT +) to a waitlist condition. CA-CBT + is based on the cultural adaptions of Cognitive Behavioural Therapy (CBT) by Hinton [51] and was adapted to the Afghan culture. The sessions covered the topics psychoeducation, emotional distancing and regulation techniques, mindfulness and relaxation techniques, anxiety, trauma recall and anger protocols, working with cognitions, somatic complaints and cultural syndromes. In contrast to the other studies with a control group design, which have a sufficient number of subjects (> 130 participants), Kananian et al. [46] included only 25 participants.

An RCT with care as usual as a control condition was conducted by Wiechers et al. [39] in Germany with refugees who were native-speakers of Arabic or Dari/Farsi and/or fluent in English or German. The intervention Empowerment group therapy for refugees (Empowerment) is based on cognitive behavioral therapy and comprises 16 sessions, each starting with a breathing exercise. Sessions 1–5 focus on psychoeducation and behavioral activation in the context of displacement. A culturally sensitive explanatory model is developed. Sessions 6–10 impart coping skills in dealing with migration-related acute stress, disturbed sleep, and somatic pain. Sessions 11–14 focus on emotion regulation strategies. In the final two sessions, information about further treatment options within the German mental health system is given.

Tay et al. [34] compared Integrative Adapt Therapy (IAT) to manualized CBT. Lay counsellors delivered IAT under supervision. The aim was to help refugees trace their emotional and behavioral problems to the underlying psychosocial disruptions. IAT is based on the Adaption and Development After Persecution and Trauma (ADAPT) model [52], which identifies five key psychosocial systems that support mental health in stable societies but are undermined by the refugee experience: safety/security, attachments, justice, role/identity disruptions, existential meaning. Seven treatment strategies (skills) were trained: psychoeducation, trauma narrative/modified exposure, problem solving, stress management, emotional regulation, cognitive reappraisal, meaning making [34].

Acarturk et al. [38] examined group gPM + for Syrian refugees in Türkiye against care as usual. The treatment consisted of 5 weekly group sessions, each of 2 h, delivered by non-specialists. For a description of the content of the sessions, see 3.2.1.1.

#### Digital intervention

The digital intervention Step-by-Step was based on behavioral activation, guided by nonspecialized helpers [36]. In five-sessions on an internet-connected device psychoeducation and training in behavioral activation were mediated through illustrated narratives. Therapeutic techniques such as stress management, a gratitude exercise, positive self-talk, strengthening social support, and relapse intervention were introduced. The narratives could be adapted to the user. The tool has a female and male version, each with two versions for different living situations (married with children or unmarried) and participants can choose the appearance of the character. Patients could use their phone or a computer in the camp during the trial. Cuijpers et al. [36] compared Step by Step with enhanced care as usual (ECAU). ECAU consisted of the first session of Step-by-Step, a basic psychoeducation, and a referral to evidence-based care, delivered through the app or website.

#### Summary of findings from controlled trials

Only the study by Vijayakumar et al. [41] showed no significant improvement in the symptoms of depression, however in this study the suicide rate improved significantly. Acarturk et al. [38] reported significant change only in the subgroup of those with probable baseline depression and not in the total sample. All other studies reported a significant reduction of depressive symptoms. It is notable that most of the randomized controlled trials reviewed here reported large treatment effects for reducing depressive symptoms (see Table 7). IAT was superior to CBT and specialized treatment was superior to care as usual. Apart from Tay et al. [34] the trials did not include credible control conditions, which hinders any conclusion about the efficacy of non-specific factors. CETA, Empowerment and the digital intervention Step-by-Step had high dropout rates (21%, 47% and 46%), indicating that while the interventions seems effective, they may not be well accepted. The studies varied in content and the extent to which various components were applied. The absence of treatment fidelity checks adds to the difficulty in disaggregating the specific components of effective treatment. This highlights the need for more investigation into treatment efficacy, the mechanisms of change, as well the acceptance of treatment methods amongst refugees before recommendations can be made.

**Table 7:** Effectiveness of the quantitative randomized trials included (k = 9)

			**Between-groups (IG vs. CG)**			**Within-group (Pre to Post)**		
**First author (year)**	**Instrument**	**Analysis**	**Significant symptom reduction / Significant Mean difference**	**Clinically reliable change/ remission**	**Effect size**	**Significant symptom reduction/ Mean difference (SD)**	**Clinically reliable change/ remission**	**Effect-size**
**Acarturk (2024) **[38]	HSCL-25,	ITT,	No in total, but Yes in subgroup analyses of baseline depression; Subgroup Pre to Post: difference:−0.17; CI: −0.32,−0.02); p < 0.028** Follow up 3-months: Not significant	NA for depression only	d = 0.27 (small)	NA	NA	NA
**Bolton (2014) **[40]	HSCL-25, adapted version	ITT	Yes difference, −0.49 (95% CI: −0.59,−0.40); P < 0.001***)	NA	HSCL −25 d = 1.16 (large); (d = 1.44 (large) for sub-population with severe depression)	IG: yes; Mean Pre score: NA; Mean Post Score: NA; difference: −1.02; 95% CI: −1.12, −0.91; p: NA) CG: yes; Mean Pre score: NA; Mean Post Score: NA; difference −0.52; 95% CI: −0.63; −0.42; p: NA)	NA	NA
**Cuijpers (2022) **[36]	PHQ-9	ITT/Completers	Yes Pre to Post: b = −2.81; SE = 0.66; p < 0.001) Follow up 3-months: b = −3.63; SE = 0.72, p < 0.001*** (regression models)	Yes IG: 37.1% response; 20.8% complete remission CG: 13.3% response; 4.9% complete remission p < 0.001*** Odd Ratio: *ITT* Response: 3.8 Complete Remission: 5.1 *Completers* Response: 4.4 Complete remission: 6.6 NNT: *ITT* Response: 4 Complete Remission: 6 *Completers* Response:4 Complete remission: 6	Pre to Post: g = 0.48; 95% CI: 0.26;0.70 (moderate) Pre to Follow up (3 months): g = −4.28; 95% CI:0.22;0.68 (moderate to large)	NA	Yes 37.1% response; 20.8% complete remission in IG	NA
**De Graaff (2023) **[35]	HSCL-25	ITT/ Completers	Yes difference, −0.34 (95%-CI: −0.486 to −0.199; p < 0.0001) by Mixed-model analyses (Mma); −0.23 by covariate-adjusted mixed-model analysis difference: −0.34; 3-month follow up: Mean follow up Score: 1.91, difference −0.28; 95%- CI = −0.421 to −0.131; p = 0.0002 (Mma)	NNT = 4.2 (risk difference: −0.24; 95% CI −0.314 to −0.166); Completers only: IG Post: Recovered 0; Improved 41.2% Follow-up: Recovered 2.4%, Improved: 38.1%) CG Post: Recovered 1.1%; Improved 16.1% Follow-up: Recovered 0%, Improved: 23.9%)	Pre-Post: d: 0.50 (0.43 covariate adjusted) Post-follow up (3month): d = 0.42 (Larger effect size for participants wo scored above cut-off at baseline for depression (p < 0.0001 and p > 0.0001 follow up))	NA	NA	NA
**Kananian (2020) **[46]	GHQ-28; PHQ-9, Farsi Versions	ITT	Yes GHQ-28: F(1,22) = 29.16, p < .001; GHQ-28 subscale depression, p < .001***; PHQ-9: F(1,22) = 1.18, p < .001***	Yes A) Post treatment: 100% response rate (GHQ-score decrease of 5) of IG patients and 8.3% of CG patients; difference was highly significant, p.005 B) 12-months follow up: response rate of 58.3% in IG	Pre to Post GHQ-28 d = 3.0 (large); PHQ-9 d = 1.5 (large) Pre to Follow up (12 months post treatment) GHQ-28 d = 1.0 (large), PHQ-9 d = 0.6 (moderate)	NA	NA	NA
**Northwood (2020) **[43]	HSCL-25_depression	ITT	Yes difference, 5.5, 95% CI, 3.9 to7.1, p < .001***	NA	partial eta squared = 0.214 (large)	NA	NA	NA
**Tay (2020) **[34]	RMHAP, depression module	ITT	Yes IAT vs. CBT difference, -.07 (95% CI: −0.13 to −0.01, p = .020*	NA	NA	Yes IG (IAT): Mean Pre score: 1.82, 95%-CI 1.74–1.09 (NA); Mean Post Score: 1.27 95%-CI, 1.11–1.19; difference: 0.55; p < 0.05** CG (CBT): Mean Pre score: 1.82, 95%-CI 1.74–1.9 (N/A); Mean Post Score: 1.33 95%-CI, 1.28–1.38; difference: 0.49; p < 0.05**	NA	IAT: d = 1.4 (large) CBT: d = 1.11 (large)
**Wiechers (2023) **[39]	PHQ-9: MADRS	ITT	yes	Yes, significant response and remission in IG compared to CG	PHQ: d = 0.68, 95% CI 0.21–1.15) (moderate); MADRS: d = 0.51, 95% CI 0.04–0.99) (moderate)	NA	NA	NA
**Vijayakumar (2017) **[41]	CESD-R; SSI, tamil version	ITT	Depression (CESD_R): no; IG: 25% vs. 17%, p = .000; Suicidal behaviour (SSI): yes; difference 519, 95%-CI: 136 to 902, p = .01**	NA	NA	Yes for high risk of suicide IG (n = 158): Mean Pre score: 21.54 (17.3); Mean Post Score: 14.7 (19.8); difference: 0.38; CI: NA; p < 0.001***	NA	NA

*IG* Intervention Group, *CG* Control Group, *N/A* not available, *d* Cohen’s d (Cohen,1988), *g* Hedge’s g, *b* b coefficient, *ITT* Intent to Treat, *HSCL* Hopkins Symptom Checklist, *GHQ* General Health Questionnaire, *PHQ* Patient Health Questionnaire, *CES-D* Center for Epidemiological Studies, *BDI* Beck´s Depression Inventory, *RMHAP* Refugee Mental Health Assessment Package, *SSI* Beck`s Scale for Suicidal Ideation, *NNT* Number of needed to treat, *MADRS* Montgomery-Asberg Depresion Rating Scale

*p *< 0.050*

* p *< 0.010**

*p *< 0.001***

### Studies not involving a control condition

Eleven studies did not involve a control condition. Five were conducted in an individual setting and five in a group setting. The sample sizes ranged from n = 7 [47] to n = 144 [33] with the median of n = 29 participants.

#### Individual Setting

Kinzie et al. [42], Stammel et al. [48] and van Wyk et al. [50] studied multidisciplinary treatments in specialized centers. They offered culturally tailored psychotherapy, medication, counselling and social work. The interventions were diverse and patient-centered, not manualized, depending on the individual needs of the patient and on the capacities of the health professional. They treated the patients for about a year on a weekly or biweekly basis. Brakemeier et al. [45] offered Interpersonal Integrative Therapy for Refugees (IITF—abbreviated from the German term Interpersonelle Integrative Therapie für Flüchtlinge). The patients received 25 weekly sessions that focussed on modifying five treatment foci: role change, integration, interpersonal conflicts, grief, and isolation/loneliness. Pishyar et al. [37] reported on a small group of patients (n = 16) whom the author treated using the therapeutic de-reflection intervention [44]. De-reflection intervention is a logotherapy technique that aims to decrease negative self-observation by directing attention from problems and symptoms to meaningful values in the patient’s life, helping individuals to move toward self-transcendence. The therapy includes activity planning, multidimensional hygiene, a personal journey toward self-transcendence, managing negative attitudes and self-focused attention.

#### Group Setting

Berkson et al. [49] investigated the Cambodian Health Promotion Program (CHPP), a culturally tailored health education program for refugee survivors of torture, which uses handouts and videos for information transfer. Five key elements are a part of the training: 1. concept of health promotion and disease prevention, 2. nutrition, 3. physical activity, 4. stress, depression and sleep, 5. relationships. Kananian et al. [47] executed a pilot study for the CA-CBT therapy [46] but without the problem-solving sessions. Poudel-Tandukar et al. implemented an intervention called Social and Emotional Wellbeing (SEW) in two different populations. One trial was with Ukrainian and one trial with Bhutanese refugees in USA [37, 53]. It is a community based and culturally tailored multimodal intervention. Each session consists of health education, a problem-solving activity (60 min), breathing exercise and yoga (60 min). SEW has five modules: managing stress, strengthening communication skill, strengthening social networking, problem solving, creating a healthy family environment. Tay et al. [33] evaluated the previously described Integrative Adapt Therapy (see also *3.2.1.2)* within a group setting (IAT-G) during the emergency phase of a mass humanitarian crisis amongst Rohingya refugees in Bangladesh. This group intervention with five to eight participants comprised seven sessions, each lasting 90 min. Each session focused on a psychosocial support system or pillar of the ADAPT model (see 3.2.1.2). After a reflection and sharing phase in each session, CBT strategies were introduced by lay workers to deal with feelings of stress related to the disruption of the ADAPT pillars. In addition, psychoeducation and skills were mediated. The Art Based Intervention (ABI) studied by Mateos-Fernándes et al. [31] represents a further form of intervention. ABI was developed for and evaluated by a group of irregular refugees and consists of drawing, dance, musical improvisation and relaxation.

#### Summary of findings from non-controlled trials

The studies were mostly of a pilot nature with a limited number of participants. Except for the pilot study by Kananian et al. [47], all studies showed a significant improvement of depressive symptoms from pre- to post- intervention. Kinzie et al. [42], van Wyk et al. [50] and in two trials Poudel-Tandukar et al. [37, 53] even achieved remission rates of above 30%. The trial by Kananian et al. [47] showed no significant change in the Patient Health Questionnaire (PHQ) but significant symptom reduction measured by the General Health Questionnaire (GHQ-28) subscale for depression. In a later trial by Kananian et al. [46] with more participants, the reduction in the PHQ values also became significant. Note, however, that the authors allowed additional medication. Confounding variables were hardly recorded, so that the significant mean changes between pre- and post-treatment are hardly meaningful. Nevertheless, the studies provide important information for the development of interventions. It seems that the use of psychotherapy at least does not lead to a worsening of symptoms. There is therefore no risk associated with the implementation of such interventions and they were mostly accepted by the refugees. Only the trials of Brakemeier et al. [45] and Kananian et al. [47] had high dropout rates (> 20%). For more details see Table 8.

**Table 8:** Effectiveness of the non-randomized trials included (k = 11)

			**Within-group (Pre to Post)**		
First author (year)	**Instrument**	**Analysis**	**Significant symptom reduction/ Mean difference (SD)**	**Clinically reliable change/ remission**	**Effect-size**
Berkson (2014) [49]	HSCL, Cambodian version	ITT	Yes Mean Pre score: 1.99; Mean Post Score: 1.72; difference: 0.28; CI: N/A; p = 0.000***	Yes Above HSCL Treshold for DD: pre 53% to post 44%; CI: NA; p = .034*; 17% of patients reduction)	NA
Brakemeier (2017) [45]	HSCL-25, Arabic version	ITT	Yes p = NA	Yes Therapists’ third-party assessment: 19% of patients remission	d = −0.63 (moderate)
Kananian (2017) [47]	GHQ-28; PHQ-9, Farsi Versions	Completer/ ITT	PHQ: no Mean Pre Score (SD): 15.7 (6.4) Mean Post Score (SD): 11.6 (5.7); WSR: −1.4 GHQ-28 subscale severe depression: yes Mean Pre Score (SD): 9.4 (3.8); Mean Post Score (SD): 6.4 (2.2); WSR: −1.5**, p < .025	PHQ and GHQ subscale depression: NA GHQ total: no remission (GHQ < 6), reliable change of n = 5 (d > 7.5 (α = 0.05, reliability = 0.90, SD pre = 8.4)	PHQ-9: d = 0.6/0.5 (moderate); GHQ-28 Severe Depression subscale: d = 1.0/0.7 (large)
Kinzie (2012) [42]	CES-D	Completer	Yes Mean Pre score: 43; Mean Post Score: 13.5; difference: 29.5; CI: NA; p = NA p = 0.000***	Yes Score > 16 at intake (n = 21): after one year n = 9 dropped out of the depressed range, n = 11 dropped at least 10 points (30% reduction)	NA
Mateos-Fernandez (2020) [31]	BDI, French Version	ITT	Yes Mean Pre score: 5.27 (3.19); Mean Post Score: 2.54 (2.42); difference: −2.10; CI: NA; p = NA; p = .036*	Yes pre-test: n = 6 minor depressive, n = 3 moderate post-test: n = 3 minor depression, n = 1 moderate depression	Hedges`G = 0.924 (large)
Pishyar (2012) [44]	BDI	ITT	Yes Mean Pre score: 36.5 (12.35); Mean Post Score: 8.12 (2.41); difference: 28.37; CI: NA; t-value 9.12, df = 15, p < 0.001***	NA	NA
Poudel-Tandukar (2021) [53]	HSCL-25, Nepal version	ITT	Yes Mean Pre score: 26.7 (10.9); Mean Post Score: 20.8 (9.1); difference: 5.9; CI: N/A; p < 0.004**	Yes 48% above cut off for depression before intervention to 14% after the intervention (p < .001***)	d = 3.3 (large)
Poudel-Tandukar (2024) [32]	HSCL-25	ITT	Yes Mean Pre score: 29.22 (9.63); Mean Post Score: 22.93 (7.33) Difference to-t1 = 6.29 (p < 0.01	Yes, above-treshold depression reduced from 58.06% before to 22.58% after intervention (p < 0.01)	NA
Stammel (2017) [48]	HSCL-25	Completer	Yes t0 Mean Pre score: 2.8 (0.5); t1 Mean Post Score: 2.7 (0.7); t2 Mean Follow-up score: 2.2 (0.7); difference t0-t1: 0.1; t0–t2: 0.6; Intercept: 2.86***, 95%-CI: 2.0, 3.72; Time −0.04***, 95%-CI −0.05, −0.02, p < .001***	NA number of clinical cases still high after one year of treatment	Pseudo R^2^ = .28
Tay (2021) [33]	PHQ-9	Completer	Yes Mean Pre score: 17.5 (95% CI 19.7–18.3); Mean Post Score: 7.3 (95% CI 6.4–8.2) Mean Follow-up score: 6.1 3 (95% CI 5.2–7.0) difference:; CI: N/A; p < 0.05*	NA	Pro to Post d = 1.59 (medium) Pre to follow-up d = 1.9 (large)
Van Wyk (2012) [50]	HSCL-37, English version	NA	Yes Mean Pre score: 1,69 (0.5); Mean Post Score: 1.31 (0.3); difference: 0,38; CI: NA; p < 0.001***	Yes pre: 37% in clinically significant range post: 7%	r = 0.57 (very large)

*IG* Intervention Group, *CG* Control Group, *N/A* Not available, *d* cohen`s d (Cohen,1988), *ITT* Intent to Treat, *HSCL* Hopkins Symptom Checklist, *GHQ* General Health Questionnaire, *PHQ* Patient Health Questionnaire, *CES-D* Centre for Epidemiological Studies, *BDI* Beck´s Depression Inventory, *RMHAP* Refugee Mental Health Assessment Package, *SSI* Bek`s Scale for Suicidal Ideation, *WSR* Wilcoxon-Signed-Rank-Test

*p*< 0.050*

*p*< 0.010**

*p*< 0.001***

#### Methodological quality assessment of included studies

The applied quality criteria can be found in Table 9. Acarturk et al. [38]was the only study with high quality ratings. Apart from the RCT by Vijayakumar et al. [41] and Wiechers et al. [39], which were rated low, the quality of all other RCTs (k = 6) was classified as moderate. Reasons for a moderate rating were often insufficient or inadequate information about the outcome data, for example high drop-out rates [36, 39, 40]. Non-randomized studies are already of poorer quality due to their study design. But even within their category, the identified trials scored poorly with k = 8 trials categorized as low quality. No study was of high quality and k = 3 were of medium quality [42, 45, 48]. For example, k = 7 trials categorized as low quality did not consider confounding variables (see Table 9).

**Table 9:** Adapted rating of the methodological quality of included studies (k = 20) based on the Mixed Methods Appraisal Tool (MMAT) [30]

	**Randomized controlled trials**					**Non-randomized trials**					
	Randomization^a^	Groups comparable^b^	Outcome Data^c^	Blind assess^d^	Adherence^e^	Representativity^f^	Measurements^g^	Outcome data^h^	Confounders^i^	Intervention^j^	**Overall quality assessment**
**Acaturk (2024) **[39]	+	+	+	+	+						high
**Berkson (2014) **[49]						-	+	o	-	o	low
**Bolton (2014) **[40]	+	+	-	+	+						medium
**Brakemeier (2017) **[45]						+	+	-	+	o	medium
**Cuijpers (2022) **[36]	+	+	o	+	+						medium
**De Graaff (2023) **[35]	+	+	o	+	+						medium
**Kananian (2017) **[47]						-	+	-	-	o	low
**Kananian (2020) **[46]	o	+	+	+	-						medium
**Kinzie (2012) **[42]						+	+	o	+	o	medium
**Mateos-Fernandez (2020) **[31]						-	+	o	-	o	low
**Northwood (2020) **[43]	+	+	o	+	-						medium
**Pishyar (2012) **[44]						-	+	o	-	o	low
**Poudel-Tandukar (2021) **[53]						+	+	o	-	o	low
**Stammel (2017) **[48]						+	+	o	+	o	medium
**Poudel-Tandukar (2024) **[32]						+	+	o	-	o	low
**Tay (2020) **[34]	+	+	o	+	o						medium
**Tay (2021) **[33]						+	+	o	o	o	low
**Van Wyk (2012) **[50]						-	+	o	+	o	low
**Wichers (2023)**	o	+	-	+	o						low
**Vijayakumar (2017) **[41]	+	-	o	+	o						low

+ quality criterion met,—- quality criterion not met, 0 = not enough information available

Overall assessment of methodological study quality: HIGH = all criteria met, MEDIUM = 1-2 quality criteria not met / not enough information available, LOW = more than 2 quality criteria not met / not enough information available

^a^Is randomization appropriately performed?

^b^Are the groups comparable at baseline?

^c^Are there complete outcome data (complete data value >80% and Drop Out >20%)?

^d^Are outcome assessors blinded to the intervention provided?

^e^Did the participants adhere to the assigned intervention?

^f^Are the participants representative of the target population?

^g^Are measurements appropriate regarding both the outcome and intervention (or exposure)?

^h^Are there complete outcome data (complete data value >80% and Drop Out <20% post intervention)?

^i^Are the confounders accounted for in the design and at least one analysed?

^j^During the study period, is the intervention administered (or exposure occurred) as intended?

## Discussion

### Summary of key findings

Of 1316 initial hits, a total of 20 studies met our eligibility criteria. Nine of these trials were carried out in an individual setting and ten in a group setting, with one of the trials conducted digitally. Nine studies were designed as randomized controlled trials, with only one study using an active control group. Three of the trials applied multimodal treatments, and a total of sixteen studies applied manualized treatments. Overall, nineteen out of twenty trials reported a significant improvement in depressive symptoms.

Seventeen interventions in the included trials were developed in industrialized countries within a western context and were adapted to the culture or specific needs of the refugee group investigated. It would be desirable to have more trials with refugee populations being conducted in low and middle-income countries (LMICs) and developed within different cultural backgrounds. The result of the study by Tay [34] suggests that a psychological treatment that addresses multiple social and emotional stressors associated with migration in depressive patients is superior to classic cognitive behavioral therapy. [54] Also, psychotherapy without trauma confrontation seems to be effective in refugee populations with depression. Since cognitive behavioral therapy in a culturally adapted form (CA-CBT) was the best studied therapy method with large effect sizes in this population (4 RCTs found in this review), it is likely that this intervention is effective in the treatment of depression in refugees. The intervention Empowerment Group Therapy for Refugees (Empowerment) is also based on cognitive behavioural therapy and had significant treatment effects [39]. Intensive psychotherapy in combination with case management by professional helpers who have been trained in the needs of refugees seems to be superior to non-specialised treatment in a primary care setting [43]. Other psychological interventions like the Common Elements Treatment Approach [40], Integrative Adapt Therapy [33, 34], and Problem Management Plus [35, 38] may also be effective. The digital intervention Step by Step [36] ​seems particularly promising, especially in regions that are difficult to access. Since interventions carried out with the help of an interpreter have also achieved an improvement in depressive symptoms, the use of interpreters in interventions should be considered more frequently if communication is otherwise not possible.

### Methodological strengths and limitations of this review

Our systematic search strictly adhered to the previously defined PICOS criteria (see Table 1). Although the criteria were formulated as precisely as possible beforehand, there was a need for discussion in the case of several studies, particularly with regard to the inclusion criterion (IC 2.2) of focussing the intervention on depression. The interrater reliability was therefore only low to moderate. In the discussion the exclusion criterion (EC2) of focusing on PTSD was often helpful at this point. In a new review, this criterion should either be formulated more precisely or replaced by another criterion. Whether the psychiatric interventions as studied by Kinzie et al. [42] correspond to our IC regarding the psychotherapy or psychological intervention was also discussed. The decision was inclusion, since supportive psychotherapy was offered and drug-based approaches were not compared. Only drug-based approaches were excluded. Based on this review, no statement can be made as to whether any pharmacological approach is effective in this population. Parallel administration of psychotropic medications during a trial was also not an exclusion criterion in our review. The results of the individual studies may be distorted as a result.

In general, the selection of studies to be included in systematic reviews depends very much on how the criterion was previously defined. In our review, for example, trials that were not conducted 100% on refugees were excluded. This meant that studies such as Orang et al. [55], that nevertheless predominantly analysed refugees, were excluded from the analysis. Internally displaced persons are also not represented in this review. The focus was only on refugees living in exile, because this group is particularly vulnerable, as they are often survivors of extreme situations such as war or persecution on the one hand, but also have to adapt to a foreign country on the other. This leads to additional stress due to high demands and/or lifestyle restrictions (e.g. work bans) that are specific to this group. Migrants do not have a history of potential traumatic events before and during their journey to a new country. They are also more likely to have less problems in the foreign country and therefore require a different treatment focus. Internally displaced persons were not included as they have not been exposed to post-migration stress (e.g. discrimination, labor restrictions, adapting to cultural context). Due to the expected small number of studies on depression treatment, studies were also allowed in which depression was not an inclusion criterion. In accordance with our PICOS criterion it was sufficient if the number of participants with depression at recruiting was specified (in %). If the definition of the criterion had been more strictly enforced (depression as inclusion criterion, 100% participants with depression), only five studies would have been included. Moreover we did not perform a meta-analysis at this time because the included studies were considered too heterogeneous and of insufficient methodological quality for a meta-analysis.

## Conclusions

The results of the studies identified in this review indicate that psychotherapy is effective in the treatment of depression in refugees and asylum seekers. Because of the small number of trials and their limited quality, no valid recommendations can be made regarding a specific treatment or specific method. Given the high prevalence of depression in refugees and the rising number of refugees because of global crises, there is an urgent need for more investigation into effective treatment for this population. Future research also needs to meet higher research quality standards. Additionally, the therapeutic approaches need to consider cultural adaptation beyond linguistic translation and should take into account post-migration-stressors.

## Data Availability

The datasets used and/or analyzed during the current study are available from the corresponding author on reasonable request.

## Abbreviations

ABI: Art Based Intervention
BDI: Beck´s Depression Inventory
CA-CBT: Culturally Adapted Cognitive Behavioural Therapy
CA-CBT +: Culturally Adapted Cognitive Behavioral Therapy plus Problem Solving
CASP: Contact and Safety Planning
CAU: Care as usual
CETA: Common Elements Treatment Approach
CG: Control Group
CHPP: Cambodian Health Promotion Program
DD: Depressive Disorder
DSM: Diagnostic and Statistical Manual of Mental Disorders
ECAU: Enhanced care as usual
GHQ-28: General Health Questionnaire
GRT: Parallel group-randomized trial
HSCL: Hopkins Symptom Checklist
IAT: Integrative Adapt Therapy
ICD: International Statistical Classification of Diseases
IPCM: Intensive Psychotherapy and Case Management
ITT: Intent to Treat
MMAT: Mixed Method Appraisal Tool
MINI: Mini International Neuropsychiatric Interview
NA: Not available
PHQ: Patient Health Questionnaire
PICOS: Patient/Population (P), Intervention (I), Comparator (C) Outcome (O) Study design (S)
PM +: Problem Management Plus
PROSPERO: International Prospective Register of Systematic Reviews
PTSD: Posttraumatic stress disorder
RCT: Randomized controlled trials
SEW: Social and Emotional Wellbeing
WHO: World Health Organization
WHO PM +: World Health Organization Problem Management Plus
